# AGING, COGNITION, AND COMMUNITY EXPERIENCE: CARE CONTINUA AND RISK RATIOS OF DAILY LIFE WITH MCI

**DOI:** 10.1093/geroni/igac059.2085

**Published:** 2022-12-20

**Authors:** Kendra Heatwole Shank

**Affiliations:** Towson University, Towson, Maryland, United States

## Abstract

Mild cognitive impairment (MCI) affects an estimated 15-30% of the population (Sachdev et al., 2015; Ward et al., 2012). Cognitive changes directly impact participation in instrumental activities of daily living that support the ability to age in place (Thoma-Lurken et al., 2018; Riley et al., 2014). To date, little is known about these initial challenges beyond self- and caregiver report. The purpose of this research was to understand the nature of out-of-home participation for individuals with cognitive change. Community-dwelling older adults with MCI and their care partners (n=10) were recruited in a suburban region. Data were collected utilizing the “go-along” interview method (Carpiano, 2009) in combination with geospatial mapping and photography. Results included three factors that influenced ongoing community participation related to cognitive changes, including the importance of familiarity of spaces developed over time, strategies related to roadways and parking, and the use of routine to create structure and to increase the support offered by others. In addition, the go-along methodology revealed additional insights about the experiential continuum of providing and receiving care, and how enacting a ‘relative’ risk and risk perceptions impacted participation. Results of this research offer a unique view into daily life of people with MCI and suggest ways that individuals and caregivers can be advised, and their wellbeing enhanced through continued out-of-home participation. There are also multiple applications for increasing the accessibility of community spaces for cognition inclusion. Theoretical implications are also addressed, via re-framing the selection, optimization, and compensation model for considering cognitive change.

